# Frequency of light fluctuations affects tomato morphology and physiology only at extreme amplitudes

**DOI:** 10.3389/fpls.2025.1500197

**Published:** 2025-07-08

**Authors:** J. Anja Dieleman, Guido van Steekelenburg, Kees Weerheim, Elias Kaiser, Esther Meinen, Mark van Hoogdalem

**Affiliations:** ^1^ Wageningen University and Research, Business Unit Greenhouse Horticulture, Wageningen, Netherlands; ^2^ Horticulture and Product Physiology, Wageningen University and Research, Wageningen, Netherlands

**Keywords:** LED, photosynthesis, plant morphology, adaptation, dynamic lighting

## Abstract

**Introduction:**

Electricity prices can fluctuate considerably during the day due to the dependency of solar and wind energy and varying demands. Fluctuating lighting regimes might thus be economically attractive. However, only limited knowledge is available on how plants grow under fluctuating light conditions. The aims of this study were (1) to determine effects of fluctuating light intensities on plant biomass, morphology and physiology and (2) to determine whether frequency or amplitude of the fluctuations is the main determining factor of such effects.

**Methods:**

Young tomato plants were grown under fluctuating light conditions in a range of amplitudes (200/0, 175/25, 125/75 and 100/100 µmol m^-2^ s^-1^) and frequencies (several hours, 30 minutes, minutes).

**Results:**

Plants grown under extreme light fluctuations of 0/200 µmol m^-2^ s^-1^ had reduced shoot biomass, stem length, chlorophyll content and light absorption, compared to plants grown under constant light intensity. The higher the frequency of these light fluctuations, the more severe the effects. Plants responded most extremely when light fluctuated every minute between 0 and 200 µmol m^-2^ s^-1^, having the lowest shoot dry weight, chlorophyll content, leaf area and light absorption. When light fluctuations were applied every minute between 175/25 and 125/75 µmol m^-2^ s^-1^, shoot biomass and morphology were not significantly affected. Net photosynthesis rate of plants grown under 30 min light fluctuations between 200 and 0 µmol m^-2^ s^-1^ were reduced compared to constant light and light fluctuations with a smaller amplitude. Linear electron transport rates were significantly reduced for all 200/0 and 175/25 treatments compared to constant light.

**Discussion:**

These results indicate that the frequency of light fluctuations determines plant biomass, morphology and physiology only at extreme amplitudes of light fluctuations. However, when a minimum light level is maintained, the crop can integrate these light fluctuations, maintaining crop growth and development.

## 1 Introduction

Plants are sessile organisms that are exposed to continuously changing environmental conditions in nature. One of the most variable conditions is light, with light intensity and spectral composition changing on (sub-)second to minute scales, due to wind-driven leaf flutter, plant swaying, changes in sun angle and cloud cover. Plants can adapt to these conditions by altering their morphological and physiological traits, such as leaf size and orientation, stomatal behaviour and chlorophyll concentrations (Vialet-Chabrand et al., 2017; Morales and Kaiser, 2020). In protected cultivation such as greenhouses and vertical farms, light conditions can be controlled by supplementing periods of low light intensity with artificial lighting. The standard practice is to maintain relatively constant light intensities, by adding supplemental lighting when outside global radiation is below a threshold level, under the assumption that constant light conditions are better for crop growth than fluctuating conditions. In the last decade, the traditional high-pressure sodium (HPS) lighting in greenhouse horticulture is gradually being replaced by LED lighting (Paradiso and Proietti, 2022; Dieleman et al., 2019). LEDs have a number of advantages, amongst which the high efficiency in converting electricity into light, control of the light spectrum and the option to dim or switch them on and off instantly (Morrow, 2008; Bantis et al., 2018). Recently, energy prices in many parts of the world have increased considerably. Furthermore, due to the increasing availability of solar and wind energy, and the varying demand of households and industry, prices of electricity fluctuate highly throughout the day (Shinde and Amelin, 2019). Fluctuating lighting strategies that depend on variable electricity prices might thus be an economically feasible option (Kjaer et al., 2012; Afzali et al., 2021; Kaiser et al., 2024). Kjaer and Ottosen (2011) developed a control system for lighting, based on weather forecasts, electricity prices and daily photosynthesis integral (DPI). Greenhouse trials demonstrated that using this system can reduce electricity cost by 25%, but had adverse effects on flowering percentages of campanula in autumn due to low light integrals and irregular light (Kjaer et al., 2011). When the daily light integral (DLI) was comparable, plant dry weights were not affected by the dynamic control of lighting (Kjaer et al., 2012). These findings indicate that questions remain on the effects of fluctuating light conditions on crop growth and development, in relation to photoperiod, light integral and amplitude of light fluctuations, and the underlying physiological processes.

The accumulation of plant biomass is primarily determined by the rate of leaf photosynthesis and morphological traits that determine whole-plant light absorption. Most of the available literature on the effects of fluctuating light conditions on plants is focused on the instantaneous response of photosynthesis to light intensity fluctuations (Kimura et al., 2020; Long et al., 2022). Fluctuations in irradiance cause photosynthesis rates to react dynamically and decrease average photosynthesis rates compared to those under stable conditions, due to limitations introduced by lags in the irradiance-dependent regulation of processes like CO_2_ diffusion through stomata (Kimura et al., 2020), gas diffusion within the leaf, electron transport and carbon fixation (Porcar-Castell et al., 2014; Kaiser et al., 2015). Low-light adapted leaves need 5-10 minutes to reach a steady rate of net photosynthesis after switching to a higher light intensity (Kalaji et al., 2014). This time lag may affect the response of plants to fluctuations in light intensity. When subjected to a light regime mimicking natural variations in light intensity on a relatively clear day with peak intensities of 1500 μmol m^−2^ s^−1^, Arabidopsis plants had a lower photosynthetic capacity compared to constant light conditions (Vialet-Chabrand et al., 2017), associated with a lower plant dry weight. In contrast, fluctuating light conditions following a sinusoidal pattern during the day with on top of that changes in the range of 5 to 650 μmol m^−2^ s^−1^ every 5 minutes, did not affect net photosynthesis rate of young tomato plants (Zhang et al., 2020). In a comprehensive review paper, Morales and Kaiser (2020) summarized the results of 43 data sets of 6 studies and concluded that on average, fluctuating irradiance did not affect the photosynthetic capacity of leaves, expressed as light-saturated CO_2_ assimilation. However, plant biomass was significantly reduced, which might be related to the time lag in response of *A*_net_ to fluctuating light levels, and the non-linear response of *A*_net_ to irradiance, so that a larger fraction of the light is used with a lower quantum efficiency.

Next to instantaneous effects of fluctuating light conditions on photosynthesis, plants can adapt to variable light conditions structurally, by alterations in plant morphology related to light capture, such as leaf size, specific leaf area (SLA) and chlorophyll content. In an elegant study, Bhuiyan and Van Iersel (2021) exposed two varieties of lettuce plants to fluctuating light levels, where PPFD fluctuated every 15 minutes between 0/400, 40/360, 80/320, 120/280, 160/240 μmol m^−2^ s^−1^ or was kept at 200 μmol m^−2^ s^−1^, during a 16 h photoperiod. Plants grown at 0/400 formed fewer leaves and had a lower chlorophyll content compared to those grown in other treatments. Depending on the genotype, the two most extreme light fluctuations (0/400 and 40/360) resulted in lower leaf area and shoot dry weight and a higher SLA. Similar results were reported in Arabidopsis, where fluctuating light conditions resulted in reduced leaf area, lower aboveground biomass and a higher SLA, compared to constant light intensities (Vialet-Chabrand et al., 2017). On the contrary, in young tomato plants grown under light flecks of 20 s length and 1000 μmol m^−2^ s^−1^ peak intensity applied every 5 min, leaf area, leaf dry weight and shoot dry weight were not affected, although SLA was higher (Kaiser et al., 2018). When fluctuating irradiance was applied in salt stressed and non-stressed tomato plants, leaf thickness and chlorophyll content were reduced, but shoot biomass was only affected in salt stressed plants (Zhang et al., 2020). In Arabidopsis, the chlorophyll a/b ratio tended to be lower under fluctuating light conditions compared to constant light (Vialet-Chabrand et al., 2017), which is typical for shade-acclimated leaves. In summary, plants under fluctuating light conditions commonly show a decrease in plant biomass and chlorophyll content, whereas the specific leaf area increases and photosynthetic capacity seem to remain unaffected. Little is known about the underlying mechanisms that cause structural morphological changes under fluctuating light conditions. These changes might result from decreased rates of photosynthesis over a longer period of time and/or changes in light-regulated signaling pathways that control chlorophyll biosynthesis (Wang et al., 2024).

In experimental work executed so far, light fluctuations applied have largely differed in intensity and duration. Light intensity changes were applied as either short light flecks (Kaiser et al., 2018), extreme fluctuations every 15 minutes (Bhuiyan and Van Iersel, 2021) or fluctuations ranging from 30 minutes to hours (Vialet-Chabrand et al., 2017). So far, studies that combine frequency and amplitude of fluctuating light with comparable daily light integrals are lacking (Morales and Kaiser, 2020). Therefore, in this study, we have applied fluctuating light conditions to young tomato plants in a range of amplitudes (200/0, 175/25 and 125/75 µmol m^-2^ s^-1^) and frequencies (several hours, 30 minutes, minutes), while maintaining a constant DLI. All treatments were compared with a reference treatment in which light intensity was kept constant for 16 h at 100 µmol m^-2^ s^-1^. To discriminate the effects of daily light integral and photoperiod, two treatments were added with constant intensities of 200 µmol m^-2^ s^-1^ for 8 h or 16 h, with the latter one having a higher DLI. The first aim of this study was to determine the effects of fluctuating light conditions on plant biomass, morphological traits and underlying physiological traits, such as whole-plant light absorption and net photosynthesis. Furthermore, this study aimed to unravel whether frequency or amplitude of the light fluctuations was the main determining factor for the observed plant responses. Based on previously published results, we hypothesized that only extreme amplitudes of light fluctuations would reduce plant biomass, which might be aggravated by increasing the frequency of light fluctuations. We assume that this effect on plant biomass is related to underlying processes like chlorophyll content, light absorption and the rate of photosynthesis per unit leaf area. The implications of our results for the application of intelligent control strategies for assimilation lighting in protected cultivation will be discussed.

## 2 Materials and methods

### 2.1 Plant material and light treatments

Experiments were conducted in a greenhouse compartment of 9.6 m × 15 m with 12 tables, each having a ceiling of dynamic LED modules (Philips GreenPower LED production modules Dynamic, Generation 3, Signify, Eindhoven, Netherlands) in Bleiswijk, The Netherlands (52.03302, 4.5317). The LED modules were tunable in blue (B; peak at 446 nm), white (broad spectrum with large proportion of green (G) light with peak emission at 571 nm), red (R; 660 nm) and far red (FR; 730 nm). Sunlight was blocked by closing the blackout screen (LS Obscura, Ludvig Svensson, Kinna, Sweden). The greenhouse was air conditioned, allowing the realization of winter conditions throughout the year. CO_2_ (OCAP, Schiedam, The Netherlands) was supplied and greenhouse climate conditions were set according to the cultivation strategy of young tomato plants in commercial practice.

We applied 10 light treatments in two consecutive experiments according to a randomized incomplete block design. In each experiment, 6 light treatments were applied on two tables each (two replicates) under comparable environmental and initial plant conditions. Average realised temperatures were 18.9 ˚C (19.7/17.3˚C day/night) and 18.9˚C (19.6/17.4˚C day/night), air humidity deficit of 3.9 and 4.0 g/m^3^ and CO_2_ concentrations of 619 and 630 ppm, respectively for the first and second experiment. Young tomato plants (*Solanum lycopersicum* cv. Brioso, Rijk Zwaan, De Lier, The Netherlands) were obtained from a commercial nursery (23 days after sowing), with 3-4 leaves, plant length of 8 cm and an average initial shoot DW of 0.15 and 0.11 g respectively in the first and second experiment. The plants were grown in rockwool blocks (10 x 10 x 7.5 cm; Grodan, Roermond, The Netherlands) on ebb and flood tables at a plant density of 5.7 plants/m^2^. In both experiments, 6 lighting strategies were applied (**Table 1**) on 12 tables, one treatment per table in duplicate. In experiment 1, light intensity fluctuated between 0 and 200 µmol m^-2^ s^-1^ with intervals of several hours (three light periods of respectively 2, 4 and 2 hours of 200 µmol m^-2^ s^-1^, with dark periods of 4 hours in between), 30 minutes or 1 minute for 16 h. The amplitude of the 30 min fluctuations ranged between 200-0, 175-25 and 125-75 µmol m^-2^ s^-1^. All treatments were compared with a reference treatment, in which light intensity was kept constant for 16 h at 100 µmol m^-2^ s^-1^. In experiment 2, light intensity fluctuated per minute between 200-0, 175-25, 125-75 µmol m^-2^ s^-1^. To discriminate the effects of daily light integral and photoperiod, light intensity in the reference treatment was maintained at a constant intensity of 100 µmol m^-2^ s^-1^ for 16 h and two treatments were added with constant intensities of 200 µmol m^-2^ s^-1^ for 8 h or 16 h. To prevent light pollution between treatments, tables were separated by white plastic sheets. The light spectrum applied was 5% blue, 5% green and 90% red. Light intensities were set at a height of 80 cm above the table. Light treatments were applied from 6:00 until 22:00, except for the treatment Cont 200 8 h, which had a photoperiod of 8 h applied from 8:00 until 16:00. DLI was 5.76 mol m^-2^ day^-1^ for all treatments, except the treatment Cont 200 16h, which had a DLI of 11.52 mol m^-2^ day^-1^. Experiment 1 started on October 26, 2022 and experiment 2 on November 23, 2022. Both experiments lasted for 26 days and ended with a final destructive harvest.

**Table 1:** Characteristics of constant and fluctuating light treatments in this study.

Experiment	Treatment name	Light Condition	Interval	Frequency (no. of changes in light intensity per 24 h)	Maximum and minimum light intensity (µmol m^-2^ s^-1^)	Amplitude (µmol m^-2^ s^-1^)	Photoperiod (h)	DLI (mol m^-2^ day^-1^)
1 and 2	Cont 100 16h	Constant	–	1	100 – 100	0	16	5.76
1	Hour 200/0	Fluctuating	2-4-4-4-2 h	3	200 - 0	200	16	5.76
1	30 min 125/75	Fluctuating	30 min	16	125- 75	50	16	5.76
1	30 min 175/25	Fluctuating	30 min	16	175 – 25	150	16	5.76
1	30 min 200/0	Fluctuating	30 min	16	200 - 0	200	16	5.76
2	1 min 125/75	Fluctuating	1 min	960	125 – 75	50	16	5.76
2	1 min 175/25	Fluctuating	1 min	960	175 – 25	150	16	5.76
1 and 2	1 min 200/0	Fluctuating	1 min	960	200 – 0	200	16	5.76
2	Cont 200 8h	Constant	–	1	200 – 200	0	8	5.76
2	Cont 200 16h	Constant	–	1	200 - 200	0	16	11.52

### 2.2 Measurements

To determine the effects of the light treatments on the concentrations of light capturing pigments, leaf light reflection and transmission, rate of photosynthesis, fluorescence characteristics, plant morphology and total biomass production were measured.

#### 2.2.1 Chlorophyll content

Apparent chlorophyll content was determined using the MPM-100 multiple wavelength pigment meter (Opti-Sciences; Hudson, USA) that measures the transmittance at 720 nm (T720) and 850 nm (T850), and calculates apparent chlorophyll content based on the model T850/T720 -1. Per table, 2 leaflets of the uppermost fully grown leaf of 4 plants were taken for measurements.

#### 2.2.2 Leaf light absorption

In the last week of each experiment (21 days after planting), top leaflets of the uppermost fully grown leaf of 4 plants per table were sampled and placed in plastic bags with a wet tissue paper to maintain a high humidity until measurements were taken. Reflection and transmission of light of the leaflets in the range of 350-750 nm was measured in steps of 5 nm using a spectrophotometer (Lambda 950 UV/VIS, PerkinElmer, Waltham, MA, United States). Leaf light absorption was calculated as 1 – reflection – transmission. All values are presented as fraction of incoming light.

The amount of absorbed light during cultivation was calculated by multiplying the leaf light absorption by the spectral composition of the LED light in the range of 400-700 nm. By summing this up for every wavelength and dividing it by the total light intensity, the percentage of absorbed light per treatment was determined.

#### 2.2.3 Gas exchange measurements

To assess the rate of photosynthesis over time, gas exchange measurements were performed using the LI-6800 portable photosynthesis system (LI-COR Biosciences; Lincoln, USA) with the fluorescence chamber (LI-6800-01A, area 2 cm^2^) on three plants per table between 8:00 and 10:00, between 12:00 and 14:00, and between 16:00 and 18:00 h. The light source in the fluorescence chamber was set on 90% red light (peak at 625 nm) and 10% blue light (peak at 475 nm). Other environmental conditions were 300 µmol m^-2^ s^-1^ flow rate, 0.1 kPa air pressure, 8000 rpm fan speed, 60% relative humidity, 21°C air temperature and 600 ppm [CO_2_]. The uppermost fully extended leaf was clamped into the leaf chamber. After the environment and photosynthesis rate (*A*_net_) were stable, six measurements were taken within 30 seconds, which were averaged. In experiment 1, these measurements were taken for the treatments where light fluctuated every 30 min and the reference treatment, and in experiment 2 for the treatments with continuous light.

#### 2.2.4 Chlorophyll *a* fluorescence

To establish the pattern of maximum quantum efficiency of photosystem II photochemistry (*F_v_*/*F_m_)*, photosystem II operating efficiency (Φ_PSII_), non-photochemical quenching (NPQ) and photosynthetic electron transport rate (ETR) during the day, chlorophyll fluorescence measurements were taken using the WALZ MICRO-PAM Monitoring System (Walz; Effeltrich, Germany). This system has 4 measuring heads, which do not enclose the leaf, making it suited to measure fluorescence characteristics over a prolonged period (24 h) and thus allowing measuring fluorescence and therefore photosynthetic activity close to ambient greenhouse conditions. Saturating blue light pulses of 8000 µmol m^-2^ s^-1^ were applied at 15 minute intervals for a duration of 1000 ms. The 4^th^ or 5^th^ leaf from the top of the plant was measured on 11-18 days of treatment in experiment 1 and 13-20 days of treatment in experiment 2. Based on the fluorescence signal, *F_v_*/*F_m_*, Φ_PSII_ and NPQ were calculated (Equations 1–4) after Genty et al. (1989):


(1) $$F_{v}/F_{m}=\;\frac{F_{m}-F_{o}}{F_{m}}\;$$

(2) $$\Phi \text{PSII}=\frac{F_{m}^{'}-F_{s}}{F_{m}^{'}}$$

(3) $$NPQ=\frac{F_{m}-F_{m}^{'}}{F_{m}^{'}}$$

Where *F_m_* and *F_m_’* are maximum fluorescence from dark- and light adapted leaves, respectively, *F_o_* is minimum fluorescence in dark-adapted leaves, and *F_s_* is fluorescence of a light-adapted leaf under actinic light.

ETR (µmol m^-2^ s^-1^) was as:


(4) $$\text{ETR}=\;\Phi \text{PSII}\times \text{PAR}\times \frac{P_{PSII}}{P_{PSI+PSII}}\;\times \text{Abs }$$

Where $\frac{P_{PSII}}{P_{PSI+PSII}}$ accounts for the fraction of absorbed quanta at PSII, and this was assumed to be 0.5. Abs is the fraction of incident light absorbed by the leaf, which was empirically determined based on the LED light spectrum and applied in this calculation. Eight plants were measured for a period of 24 hours per repetition. To be able to determine Φ_PSII_ under stable light conditions, measurements which occurred exactly when switching between two light intensities occurred, were removed. ETR of treatments with a photoperiod less than 16 hours was normalized by multiplying the ETR by (16 divided by the photoperiod of the treatment) and displayed as ETR_n_.

#### 2.2.5 Plant morphology and biomass accumulation

After 26 days of treatment, 5 plants per table (10 plants per treatment) were harvested destructively. The number of leaves (> 4 cm), stem length, leaf area and dry weights of leaves and stem were determined. From the 6^th^ leaf counted from above (for treatments 1 min 200/0 the 4^th^ leaf), additional morphological measurements were taken: internode length above the leaf, leaf length, leaf width, petiole length, leaf area and leaf dry weight. Leaf area was determined with a leaf area meter (LI-3100, LI-COR, Lincoln, Nebraska, USA). Dry weights were measured after drying leaves and stems for at least 48 hours at 80°C. The specific leaf area (SLA) was calculated by dividing leaf area by leaf dry mass.

#### 2.2.6 Statistics

To test the hypothesis that only extreme amplitudes of light fluctuations reduce plant biomass, which might be aggravated by increasing the frequency of light fluctuations, we applied 10 light treatments (**Table 1**) in two consecutive experiments according to a randomized incomplete block design. Light treatments were defined as (1) a combination of frequency and amplitude for fluctuating light treatments and (2) a combination of light intensity and photoperiod for treatments with constant light intensities. In each experiment, 6 light treatments were applied and replicated twice. Treatments Cont 100 16 h and 1 min 200/0 were applied in both experiments (n=4) together with 4 treatments with fluctuating light conditions (n=2) (see **Table 1**). Data assessed on several plants per table (each table was considered a repetition) was averaged yielding one value per repetition. Data were analysed using REML (Restricted Maximum Likelihood) with light treatment (see **Table 1**) as fixed term and block, being the combination of experiment and repetition as random term. The assumption of normality was fulfilled in all cases. Predicted treatment means were tested with the unprotected Fisher’s LSD test for multiple comparisons to determine differences between light treatments. P-values smaller than 0.05 were considered as significantly different.

In the REML analysis, it was additionally tested whether fluctuating light conditions compared to constant light conditions (excluding the treatment Cont 200 16 h, which had a deviating DLI) could explain the effect on any of the tested response variables, and whether amplitude or frequency, including their interaction within the fluctuating light treatments, could explain these effects. For this, a model was fitted with LightCondition/(Frequency * Amplitude) as fixed term (see **Table 1**), and block, being the combination between experiment and repetition as random term. To determine whether frequency or amplitude or the combination of both determines the effects on all response variables tested, Wald tests were used to assess the significance of the fixed model terms. P-values smaller than 0.05 were considered as significant. Data were analysed using the statistical software package Genstat (22^nd^ edition, VSN International, Hempstead, UK).

## 3 Results

### 3.1 Plant morphology and biomass accumulation

**Figure 1:**
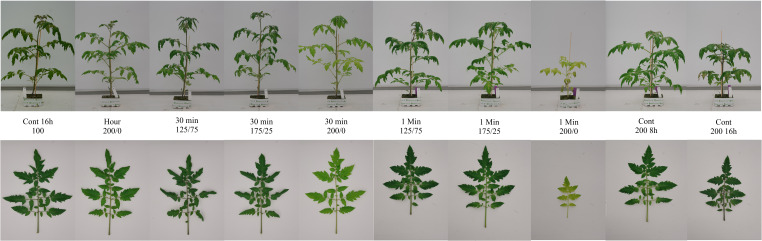
Effect of constant and fluctuating light treatments on the morphology of (top) young tomato plants and (bottom) their uppermost full-grown leaves. Treatments applied (from left to right) were constant 100 µmol m^-2^ s^-1^ during 16 h, light fluctuations every 2, 4, 4, 4 and 2 h between 200 and 0 µmol m^-2^ s^-1^, every 30 minutes between 125 and 75 µmol m^-2^ s^-1^; 175 and 25 µmol m^-2^ s^-1^; and 200 and 0 µmol m^-2^ s^-1^, every minute between 125 and 75 µmol m^-2^ s^-1^; 175 and 25 µmol m^-2^ s^-1^; and 200 and 0 µmol m^-2^ s^-1^, and constant 200 µmol m^-2^ s^-1^ during 8 and 16 h. Treatments started when plants had 3-4 leaves, pictures were taken 23 days after start of the treatments.

**Figure 2:**
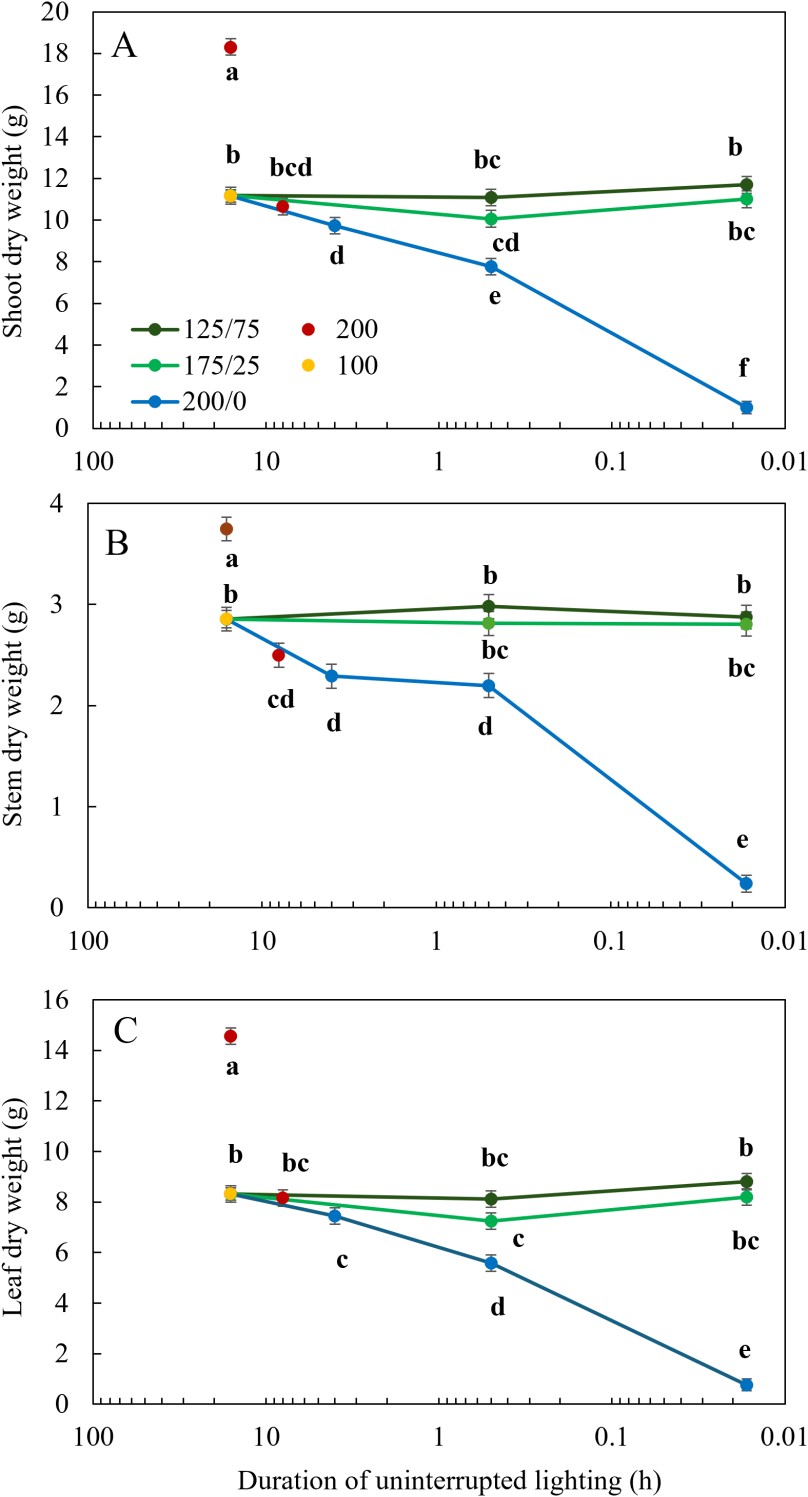
Effect of constant and fluctuating light treatments on **(A)** shoot dry weight, **(B)** stem dry weight, and **(C)** leaf dry weight of young tomato plants. Constant light treatments were applied (red symbols) during 8 h or 16 h at an intensity of 200 µmol m^-2^ s^-1^ or (yellow symbols) during 16 h at an intensity of 100 µmol m^-2^ s^-1^. Light intensities in fluctuating light treatments varied between (blue) 200 and 0 µmol m^-2^ s^-1^, (light green) 175 and 25 µmol m^-2^ s^-1^ and between (dark green) 125 and 75 µmol m^-2^ s^-1^ during 4 h, 30 min or 1 min. The duration of uninterrupted lighting, ranging from 16 h to 1 min, on the x-axis is presented as an inverse log scale. Data were collected after 26 days of treatment. Data are represented as the predicted means of 4 (treatments 100 µmol m^-2^ s^-1^ during 16 h and light fluctuations between 200 and 0 µmol m^-2^ s^-1^ every minute) or 2 (all other treatments) repetitions, each existing of 5 biological replicates ± standard error of the mean. Different letters indicate significant differences (P< 0.05).

After 26 days of light treatments, the most prominent visible effect of light fluctuations between 200 and 0 µmol m^-2^ s^-1^ was the reduced chlorophyll content of the leaves, which was progressively reduced with increasing frequency. Tomato plants subjected to 1 min changes 200/0 were significantly shorter, with lower leaf area and biomass than when they were subjected to fluctuating light with a lower frequency or a lower amplitude (**Figure 1**). Shoot dry weight was affected by fluctuating light conditions between 200 and 0 µmol m^-2^ s^-1^: the higher the frequency of these changes, the lower the shoot weight (**Figure 2A**). However, light fluctuations with an amplitude of 175 to 25 µmol m^-2^ s^-1^ or 125 to 75 µmol m^-2^ s^-1^ barely affected shoot dry weight. These relationships were supported by the significant interaction between frequency and amplitude for shoot dry weight (P< 0.001). When the same DLI was given in 8 h (200 µmol m^-2^ s^-1^) instead of 16 h (100 µmol m^-2^ s^-1^), shoot dry weight was not affected (**Figure 2A**). Increasing the DLI by providing 16 h 200 µmol m^-2^ s^-1^, resulted in an increase in shoot dry weight of 64%. Stem dry weight was significantly reduced when light fluctuated with an amplitude of 200/0 µmol m^-2^ s^-1^ in a frequency of multiple hours or 30 min compared to the treatment with a constant light intensity, and was even more strongly reduced when light was switched on/off every minute (**Figure 2B**). Stem dry weight was reduced at a photoperiod of 8 h compared to 16 h, and increased at a higher DLI (16 h 200 µmol m^-2^ s^-1^). Total leaf dry weight was not affected by light fluctuations with a an amplitude of 125/75 µmol m^-2^ s^-1^ (**Figure 2C**). Light fluctuations between 175 and 25 µmol m^-2^ s^-1^ affected leaf dry weight only when applied at a frequency of 30 min. Increasing frequencies of light fluctuations reduced leaf dry weight only at an amplitude of 200/0 µmol m^-2^ s^-1^. The interaction between frequency and amplitude was significant for stem and leaf dry weight (P< 0.001). Leaf dry weight did not differ between photoperiods of 8 or 16 h when DLI was not affected. Doubling the DLI (16 h 200 µmol m^-2^ s^-1^) increased leaf dry weight by 75% (**Figure 2C**).

When plants were subjected to fluctuations in light intensity between 200 and 0 µmol m^-2^ s^-1^ every minute, their rate of development was significantly reduced (**Table 2**), and leaves became pale and wilted (**Figure 1**). This resulted in a reduced number of leaves, leaf length, width and area, petiole length and stem length, and an increased SLA at the final destructive harvest (**Table 2**). However, when the amplitude of 1 min light fluctuations was less strong, these characteristics did not differ significantly from the treatment with constant light intensity (Cont 100 16 h). Light fluctuations with a frequency of 30 minutes hardly affected plant morphology (**Table 2**). Only leaf width and leaf area of the treatment 30 min 175/25 were lower than the treatment with constant light intensity, while all other traits were not significantly different. For all morphological traits mentioned, the interaction between frequency and amplitude of the fluctuating light conditions was significant. Plant morphology was not affected by photoperiod when DLI was maintained. Increasing the DLI resulted in a lower SLA, but did not affect other morphological traits significantly (**Table 2**).

**Table 2:** Effects of constant and fluctuating light treatments on number of leaves, leaf area and stem length per plant, and internode length, petiole length, leaf length, leaf width, and SLA of the uppermost full-grown leaf.

Treatment	Number of leaves (-)	Leaf area (m^2^)	Stemlength (cm)	Internode length (cm)	Petiole length (cm)	Leaf length (cm)	Leaf width (cm)	SLA (m^2^ kg^-1^)
Cont 16h 100	13.4 ± 0.2 ab	0.21 ± 0.01 abc	63.9 ± 3.2 abc	5.4 ± 0.3 ab	8.6 ± 0.6 ab	30.1 ± 1.1 ab	30.3 ± 1.2 abc	26.4 ± 4.7 b
Hour 200/0	13.2 ± 0.3 ab	0.19 ± 0.01 bcd	54.7 ± 4.3 c	4.9 ± 0.4 ab	8.4 ± 0.7 ab	28.7 ± 1.5 ab	26.8 ± 1.7 bcd	30.2 ± 5.8 b
30 min 125/75	13.0 ± 0.3 ab	0.19 ± 0.01 bcd	66.5 ± 4.3 ab	5.8 ± 0.4 a	8.5 ± 0.7 ab	29.0 ± 1.5 ab	28.1 ± 1.7 abcd	29.0 ± 5.8 b
30 min 175/25	13.0 ± 0.3 ab	0.17 ± 0.01 d	66.4 ± 4.3 ab	5.8 ± 0.4 a	8.1 ± 0.7 b	28.3 ± 1.5 b	25.2 ± 1.7 d	29.4 ± 5.8 b
30 min 200/0	12.8 ± 0.3 b	0.18 ± 0.01 cd	61.9 ± 4.3 abc	5.4 ± 0.4 ab	8.6 ± 0.7 ab	28.4 ± 1.5 b	25.8 ± 1.7 cd	30.1 ± 5.8 b
1 min 125/75	13.7 ± 0.3 a	0.25 ± 0.01 a	63.9 ± 4.3 abc	5.2 ± 0.4 ab	8.1 ± 0.7 b	32.9 ± 1.5 a	32.6 ± 1.7 a	25.0 ± 5.8 b
1 min 175/25	13.6 ± 0.3 ab	0.24 ± 0.01 a	69.1 ± 4.3 a	5.8 ± 0.4 a	9.5 ± 0.7 a	31.2 ± 1.5 ab	31.8 ± 1.7 ab	24.5 ± 5.8 b
1 min 200/0	9.6 ± 0.2 c	0.02 ± 0.01 e	32.3 ± 3.2 d	2.5 ± 0.3 c	5.4 ± 0.6 c	13.0 ± 1.1 c	11.0 ± 1.2 e	74.2 ± 4.7 a
Cont 200 8h	13.5 ± 0.3 ab	0.24 ± 0.01 a	62.7 ± 4.3 abc	4.9 ± 0.4 ab	9.2 ± 0.7 ab	32.6 ± 1.5 ab	33.0 ± 1.7 a	27.2 ± 5.8 b
Cont 200 16h	13.5 ± 0.3 ab	0.23 ± 0.01 ab	56.6 ± 4.3 bc	4.3 ± 0.4 b	8.5 ± 0.7 ab	30.7 ± 1.5 ab	31.8 ± 1.7 ab	10.8 ± 5.8 c

Treatments applied were constant 100 µmol m^-2^ s^-1^ during 16 h, light fluctuations every 2, 4, 4, 4 and 2 h between 200 and 0 µmol m^-2^ s^-1^, every 30 minutes between 125 and 75 µmol m^-2^ s^-1^; 175 and 25 µmol m^-2^ s^-1^; and 200 and 0 µmol m^-2^ s^-1^, every minute between 125 and 75 µmol m^-2^ s^-1^; 175 and 25 µmol m^-2^ s^-1^; and 200 and 0 µmol m^-2^ s^-1^, and constant 200 µmol m^-2^ s^-1^ during 8 and 16 h. Data are represented as the predicted means of two or 4 repetitions, each existing of 5 biological replicates ± standard error of the mean. Different letters within columns indicate significant differences (P< 0.05).

### 3.2 Chlorophyll content and light absorption

**Figure 3:**
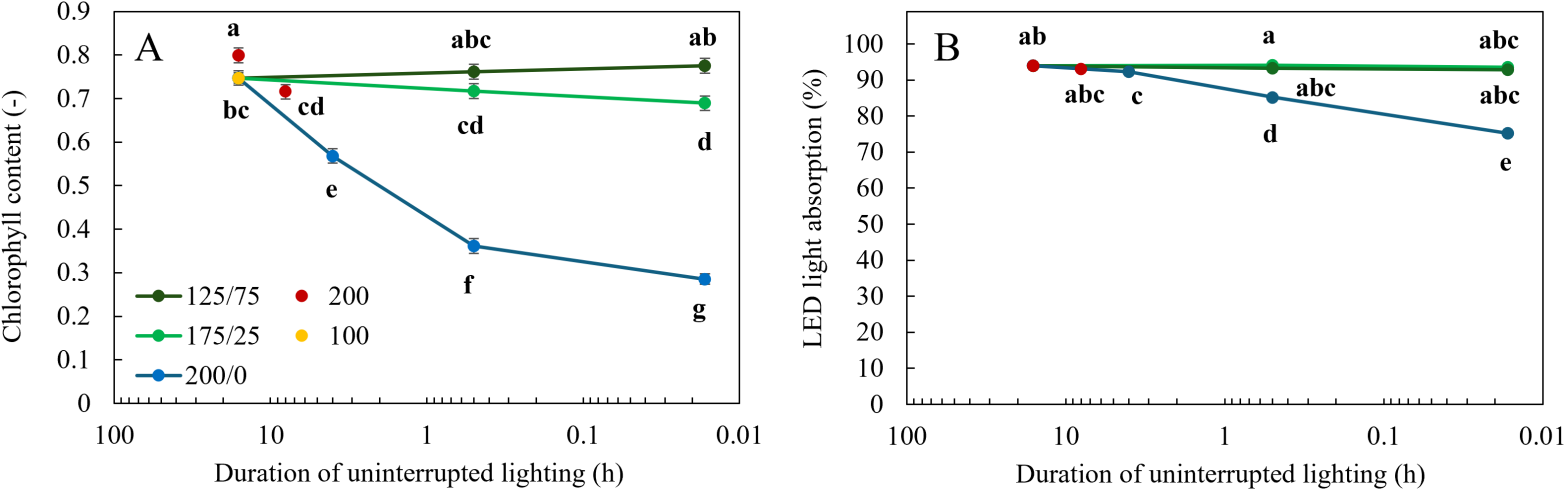
Effect of constant and fluctuating light treatments on **(A)** apparent chlorophyll content determined by MPM-100 multiple wavelength pigment meter and **(B)** calculated LED light absorption (light absorption multiplied by LED spectrum) of leaves of young tomato plants. Constant light treatments were applied (red symbols) during 8 h or 16 h at an intensity of 200 µmol m^-2^ s^-1^ or (yellow symbols) during 16 h at an intensity of 100 µmol m^-2^ s^-1^. Light intensities in fluctuating light treatments varied between (blue) 200 and 0 µmol m^-2^ s^-1^, (light green) 175 and 25 µmol m^-2^ s^-1^ and between (dark green) 125 and 75 µmol m^-2^ s^-1^ during 4 h, 30 min or 1 min. The duration of uninterrupted lighting, ranging from 16 h to 1 min, on the x-axis is presented as an inverse log scale. Data were collected after 21 days of treatment. Data are represented as the predicted means of 4 (treatments 100 µmol m^-2^ s^-1^ during 16 h and light fluctuations between 200 and 0 µmol m^-2^ s^-1^ every minute) or 2 (all other treatments) repetitions, each existing of 3-5 biological replicates ± standard error of the mean. Different letters indicate significant differences (P< 0.05).

Apparent chlorophyll content was not affected by light fluctuations between 125 and 75 µmol m^-2^ s^-1^ (**Figure 3A**). When light fluctuated between 175 and 25 µmol m^-2^ s^-1^ every minute, chlorophyll content was significantly reduced compared to a constant light intensity. At a larger amplitude, 200/0, a higher frequency of light fluctuations resulted in significantly lower apparent chlorophyll content. A higher DLI (Cont 200 16 h) resulted in a higher chlorophyll content compared to the two other treatments with constant light levels (**Figure 3A**).

**Figure 4:**
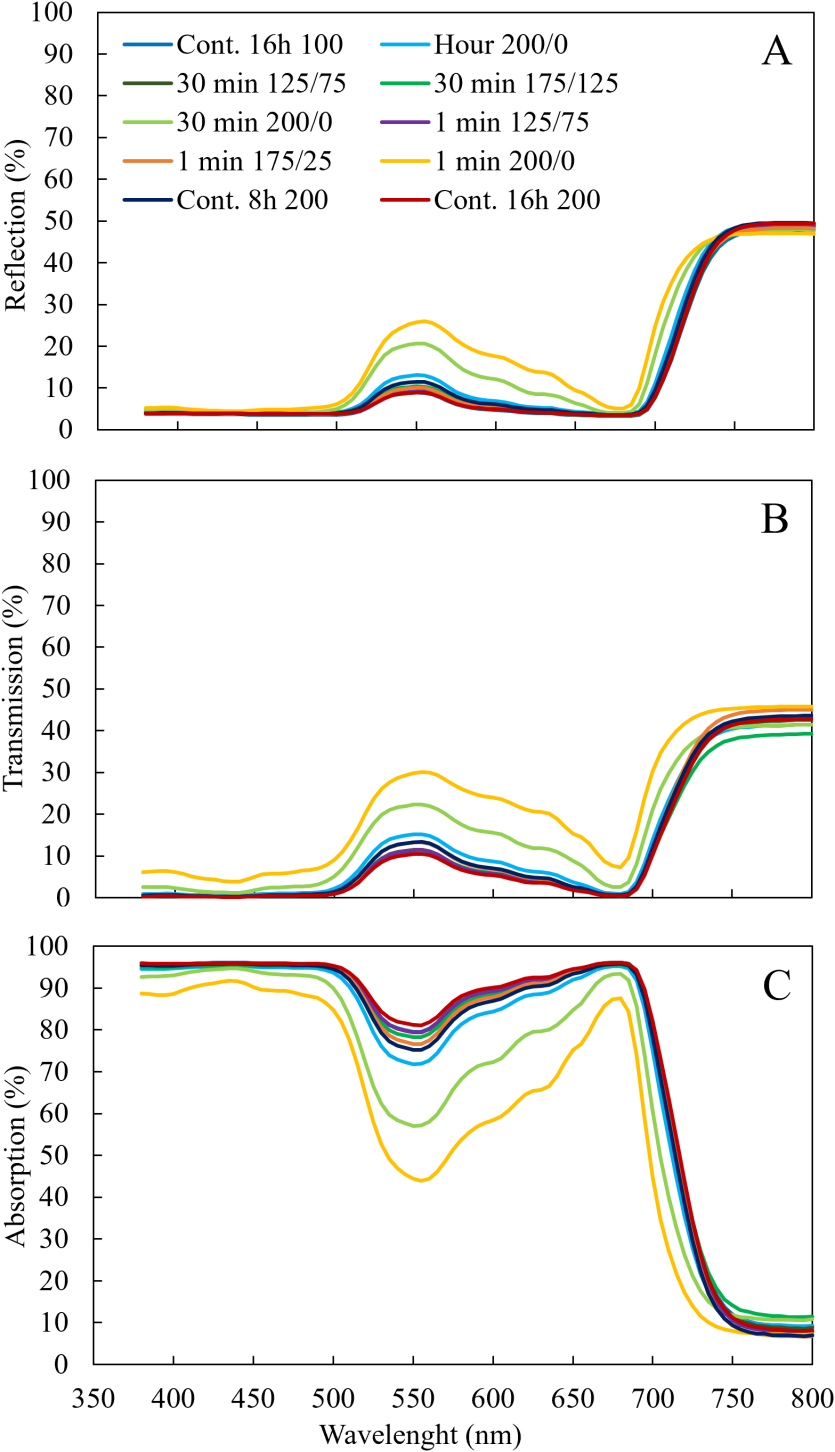
Effect of constant and fluctuating light treatments on light **(A)** reflection, **(B)** transmission measured by Lambda 950 UV/VIS spectrophotometer from 380 to 800 nm, and **(C)** absorption of leaves of young tomato plants. Leaf light absorption was calculated as 1 – reflection – transmission. Data are means of 4 (treatments 100 µmol m^-2^ s^-1^ during 16 h and light fluctuations between 200 and 0 µmol m^-2^ s^-1^ every minute) or 2 (all other treatments) repetitions, each existing of 3 biological replicates.

Plants grown under the treatments where light fluctuated between 200 and 0 µmol m^-2^ s^-1^ had the highest reflection (**Figure 4A**) in the wavelengths between 500 and 750 nm and the highest transmission between 380 and 750 nm (**Figure 4B**), resulting in the lowest leaf light absorption (**Figure 4C**). At higher frequencies of light fluctuations, these effects were more severe. The percentage of light absorption of the LED light spectrum the leaves received was not affected by light fluctuations with an amplitude of 125/75 and 175/25 µmol m^-2^ s^-1^ (**Figure 3B**). When light fluctuated between 200 and 0 µmol m^-2^ s^-1^, the fraction of LED light absorbed by the leaves was significantly reduced compared to the treatments with constant light intensity. With increasing frequency of light fluctuations, this reduction was significantly stronger (**Figure 3B**).

### 3.3 Chlorophyll *a* fluorescence and photosynthesis

**Figure 5:**
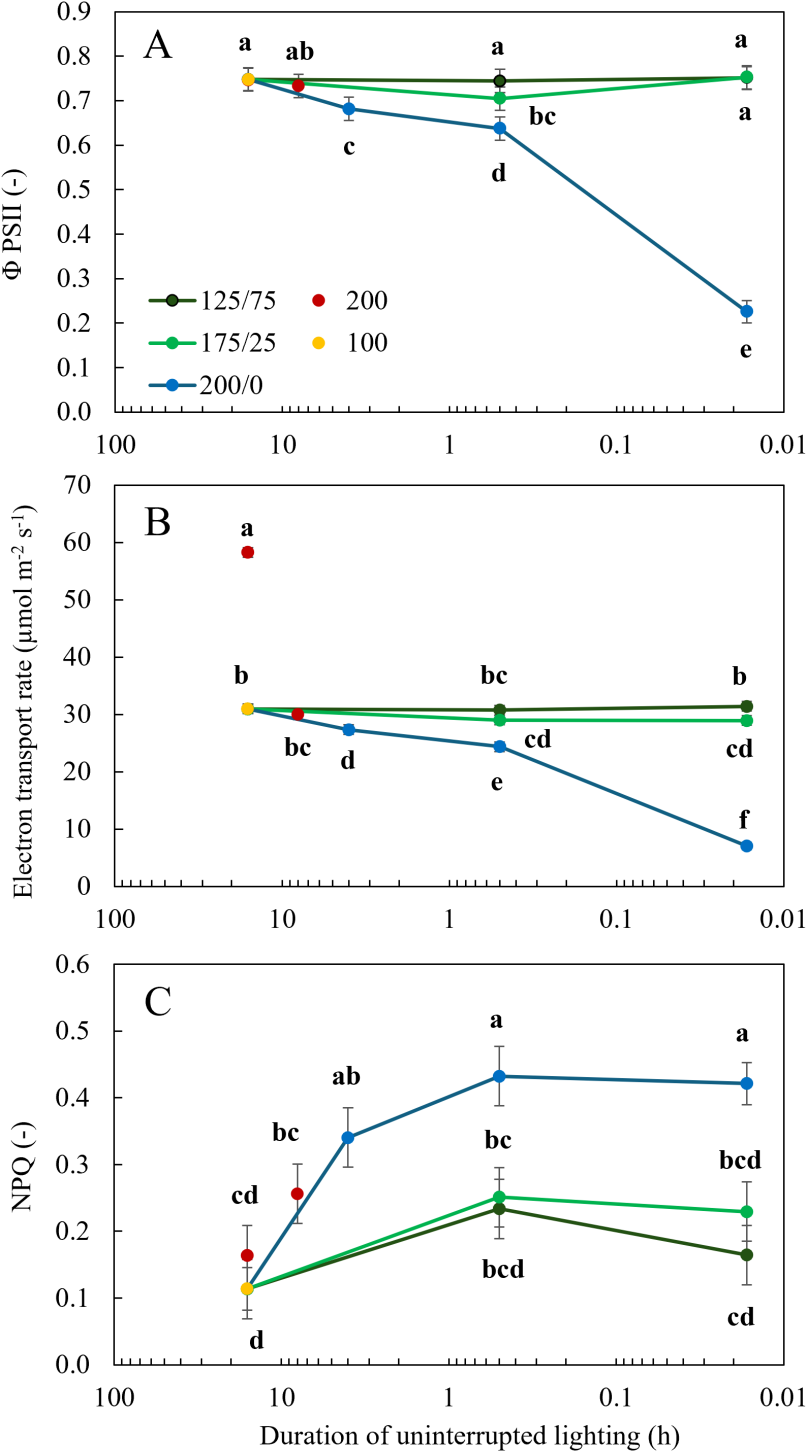
Effect of constant and fluctuating light treatments on **(A)** the efficiency of photosystem II (Φ_PSII_), **(B)** electron transport rate (ETR_n_, the average ETR normalized for the duration of the light period) and **(C)** non-photochemical quenching (NPQ) calculated based on chlorophyll fluorescence measurements using the WALZ MICRO-PAM Monitoring System of leaves of young tomato plants. Constant light treatments were applied (red symbols) during 8 h or 16 h at an intensity of 200 µmol m^-2^ s^-1^ or (yellow symbols) during 16 h at an intensity of 100 µmol m^-2^ s^-1^. Light intensities in fluctuating light treatments varied between (blue) 200 and 0 µmol m^-2^ s^-1^, (light green) 175 and 25 µmol m^-2^ s^-1^ and between (dark green) 125 and 75 µmol m^-2^ s^-1^ during 4 h, 30 min or 1 min. The duration of uninterrupted lighting, ranging from 16 h to 1 min, on the x-axis is presented as an inverse log scale. Data are represented as the predicted means of 4 (treatments 100 µmol m^-2^ s^-1^ during 16 h and light fluctuations between 200 and 0 µmol m^-2^ s^-1^ every minute) or 2 (all other treatments) repetitions, each existing of 3 biological replicates ± standard error of the mean. Different letters indicate significant differences (P< 0.05).

Dark-adapted *F_v_*/*F_m_* was 0.8 for all treatments except the treatment with 1 minute 200/0 µmol m^-2^ s^-1^ fluctuations which had a significantly reduced *F_v_*/*F_m_*, of only 0.3 (**Supplementary Figures 1**, **2**). Under actinic light, Φ_PSII_ and ETR_n_ were not affected by light fluctuations between 125 and 75 µmol m^-2^ s^-1^, irrespective of the frequency of these changes (**Figures 5A, B**). When the amplitude of light fluctuations increased to 175/25 µmol m^-2^ s^-1^, Φ_PSII_ was significantly reduced at a frequency of 30 min changes, but not when the light intensity fluctuated every minute (**Figure 5A**). At this amplitude of 175/25 µmol m^-2^ s^-1^, ETR_n_ was significantly lower than the treatment with a constant light intensity only when the light changed every minute (**Figure 5B**). When light fluctuated between 200 and 0 µmol m^-2^ s^-1^, Φ_PSII_ and ETR_n_ were significantly reduced compared to the treatments with constant light intensity. With increasing frequency of light fluctuations, this reduction was more severe (**Figures 5A, B**). The statistical model showed that for Φ_PSII_ and ETR_n_ the interaction between frequency and amplitude was significant (P< 0.001). Φ_PSII_ was not significantly affected by the photoperiod or intensity in the treatments with constant light intensity, resulting in a higher ETR_n_ in the treatment with a higher DLI (16 h 200) (**Figure 5B**). When light intensities fluctuated between 200 and 0 µmol m^-2^ s^-1^, NPQ was significantly higher than at a constant light intensity of 100 µmol m^-2^ s^-1^ (**Figure 5C**). Interestingly, NPQ was not elevated in the 200/0 1 min treatment compared to other treatments with fluctuations between 200 and 0 µmol m^-2^ s^-1^ (**Figure 5C**), despite massive reductions in dark-adapted F_v_/F_m_ in 200/0 1 min treatment (**Supplementary Figures S1**, **S2**). When a minimum light intensity of 25 µmol m^-2^ s^-1^ was maintained, NPQ did not differ significantly from treatments with constant light intensity.

**Figure 6:**
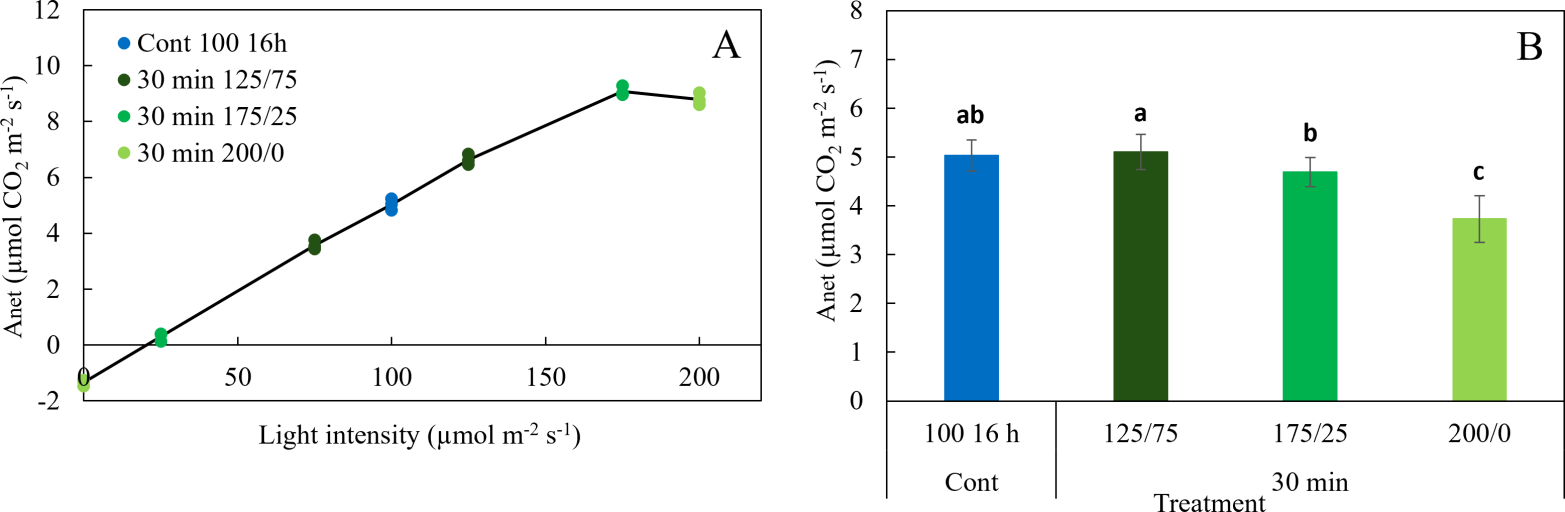
Effect of constant and fluctuating light treatments (experiment 1) on **(A)** net photosynthesis rates (*A*_net_) of leaves of young tomato plants as a function of light intensity and **(B)** net photosynthesis rate averaged over the light intensities of the treatments applied. Symbols represent measurements taken during the day (8:00 - 10:00, 12:00 - 14:00 and 16:00 -18:00 h). Data are means of 2 repetitions, each existing of 3-5 biological replicates. Different letters indicate significant differences (P< 0.05).

In experiment 1, operational net photosynthesis rate (*A*_net_) was measured under treatment conditions between 8:00-10:00, 12:00-14:00 and 16:00-18:00 in the treatments with constant light and in those where light intensity changed every 30 minutes. *A*_net_ did not differ significantly between these time frames, indicating that operational *A*_net_ remained constant during the photoperiod (data not shown). *A*_net_ increased linearly with increasing light intensities up to 125 µmol m^-2^ s^-1^ but did not increase further above 175 µmol m^-2^ s^-1^ (**Figure 6A**). This resulted in a decrease in average *A*_net_ with increasing amplitude of light fluctuations (**Figure 6B**). *A*_net_ in the treatments with 30 min fluctuations between 125 and 75, and between 175 and 25 µmol m^-2^ s^-1^ did not differ significantly from constant 100 µmol m^-2^ s^-1^, while the 30 min 200/0 treatments had significantly lower *A*_net_. Increasing the light intensity from 100 to 200 µmol m^-2^ s^-1^ in the treatments Cont 200 16 h and Cont 200 8 h almost doubled *A*_net_ with an increase of 95%,independent of the photoperiod (data not shown).

## 4 Discussion

In this study, effects of fluctuating light conditions with a range of amplitudes (between 0 and 200 µmol m^-2^ s^-1^) and frequencies (from several hours to minutes) on biomass accumulation, morphological traits and physiology of young tomato plants were quantified. Biomass was hardly affected by light fluctuations of hours, 30 minutes or minutes, as long as a minimum light level of 25 µmol m^-2^ s^-1^ was maintained. Increasing the frequency of light fluctuations further amplifies the decrease in biomass only at an extreme amplitude of 200/0 µmol m^-2^ s^-1^. The observations underlying these conclusions and the implications of these results are discussed below.

### 4.1 Fluctuating light conditions affect biomass accumulation via light absorption and photosynthesis

When young tomato plants were exposed to fluctuating light conditions, shoot dry weight was reduced,. The higher the frequency of light fluctuations between 200 and 0 μmol m^−2^ s^−1^, the more severely biomass was reduced (**Figure 2A**). When a minimum light level of 25 μmol m^−2^ s^−1^ was maintained, plant biomass was barely affected. These results are in agreement with a study where lettuce plants were exposed to 15 minutes light fluctuations in the range of 0/400 compared to constant 200 μmol m^−2^ s^−1^ (Bhuiyan and Van Iersel, 2021), where only the most extreme light fluctuations (0/400 and 40/360 μmol m^−2^ s^−1^) resulted in lower shoot dry weight, due to reductions in chlorophyll content and *A*_net_. In Arabidopsis, light fluctuations (up to 1500 μmol m^−2^ s^−1^) mimicking natural variation in sunlight also reduced aboveground biomass, as well as leaf area, leaf thickness and light absorption (Vialet-Chabrand et al., 2017), but did not affect *A*_net_. Most morphological traits such as stem length and leaf length were only significantly reduced at 1 min 200/0 light fluctuations, although leaf width and leaf area were also significantly reduced in the treatment 30 min 175/25 (**Table 2**), but not in 1 min 175/25. Surprisingly, leaf area was higher at 1 min light fluctuations 175/25 and 125/75 than at 30 min fluctuations at the same amplitudes, which could not be explained by differences in leaf length, and only for 175/25 by leaf width. SLA increased under fluctuating light conditions between 200 and 0 μmol m^−2^ s^−1^ every minute (**Table 2**), which agrees with findings of Bhuiyan and Van Iersel (2021) and Kaiser et al. (2018). The chlorophyll content showed a strong negative response to light fluctuations, in agreement with previous studies (Kjaer and Ottosen, 2011; Zhang et al., 2020; Bhuiyan and Van Iersel, 2021). With increasing frequency of light fluctuations, the reduction in chlorophyll content was stronger (**Figure 3A**). Leaf light absorption is mediated by photosynthetic pigments (chlorophylls and carotenoids), which capture light to power the light reactions of photosynthesis. Since chlorophyll *a* and *b* are the primary photosynthetic pigments (and carotenoids serve as accessory pigments) with absorption peaks in blue and red light, leaf light absorbance was expected to differ between treatments, given the differences in apparent chlorophyll content (**Figure 3A**). Indeed, leaf light absorption was reduced when light fluctuated between 200 and 0 μmol m^−2^ s^−1^ (**Figure 4C**), primarily in 500-600 nm (green) and 600-700 nm (red) regions. Reflectance between 400 and 500 nm was not affected by the light treatments applied, in spite of differences in chlorophyll content, suggesting that this reflectance value is suited as reference in vegetation indices (Gitelson and Merzlyak, 1996). LED light absorption was reduced in the 200/0 treatments (**Figure 3B**), more severe so at higher frequencies of light fluctuations. Since the LED light spectrum primarily consisted of red light (90%), leaf light absorption was primarily affected by the treatment effects on light absorption between 600 and 700 nm. LED light absorption was 93% in the treatment with constant 100 μmol m^−2^ s^−1^ and 85% in the treatment 30 min 200/0, which is a reduction of 9%. Since leaf area did not differ significantly between these two treatments (**Table 2**), total plant light absorption may have been reduced, assuming similar light extinction patterns in these plants. However, shoot biomass in the treatment with constant 100 μmol m^−2^ s^−1^ was 30% higher than in the treatment 30 min 200/0 (**Figure 2A**), indicating that leaf light absorption can only be a part of the explanation for the difference in biomass.

Morales and Kaiser (2020) suggested that the reduction in plant biomass under fluctuating light conditions might be related to, amongst others, the non-linear response of *A*_net_ to irradiance. Indeed, our measurements show that *A*_net_ increased linearly up to 125 μmol m^−2^ s^−1^, and levelled off at higher light intensities. Surprisingly, *A*_net_ did not increase between light intensities of 175 and 200 μmol m^−2^ s^−1^ (**Figure 6A**), resulting in reduced rates of photosynthesis in the treatments where light intensities fluctuated between 175/25 and 200/0 μmol m^−2^ s^−1^ (**Figure 6B**). Large light intensity fluctuations thus negatively affected the photosynthetic performance of tomato leaves, which corresponds to the findings of Bhuiyan and Van Iersel (2021) in lettuce. The reduction of *A*_net_ correlated with the observed reduced shoot dry weight. In the treatments 30 min 175/25 and 200/0 μmol m^−2^ s^−1^ in experiment 1, shoot dry weight was reduced by 7 and 26%, respectively, compared to constant light, whereas their *A*_net_ was reduced by 7 and 28% respectively. When light fluctuations of 30 min were applied, dark adapted F_v_/F_m_ was not affected, indicating that these treatments did not affect the intactness of photosystem II reaction centres. When light intensities fluctuated between 175/25 and 200/0 μmol m^−2^ s^−1^ every 30 min and every minute, Φ_PSII_ was reduced, which is likely due to slow photosynthetic induction (Kaiser et al., 2015). Similar results were obtained in several Arabidopsis accessions that showed reduced biomass under fluctuating light, which strongly correlated with a reduction in Φ_PSII_ (Kaiser et al., 2020). Calculated ETR (**Figure 5B**) was reduced at 30 min 200/0, which corresponds to *A*_net_ measurements (**Figure 6**). Non-photochemical quenching (NPQ) is typically upregulated under stressful conditions, and its relaxation after a high-to-low irradiance transition can be relatively slow (Zhu et al., 2004). Directly after such irradiance transitions, NPQ competes with photochemistry, thereby reducing the energy available for photosynthesis, and this could have reduced photosynthesis during the short periods (1 min) of 200 µmol m^-2^ s^-1^ available to the system. Indeed, Φ_PSII_ and ETR were very low when measured in these leaves (**Figures 5A, B**), which may have been caused by high NPQ under these conditions (**Figure 5C**). We observed that NPQ was generally highest in 200/0 treatments, regardless of the frequency of light fluctuations (**Figure 5C**). This suggests that intermittent darkness was more stressful than intermittent periods of low light intensity. At the same time, dark-adapted F_v_/Fm was mostly unchanged between treatments (**Supplementary Figures S1**, **S2**), suggesting that upregulated NPQ in the 200/0 treatments was effective in protecting photosystem II from excitation pressure (except for the 200/0 1 min treatment, in which F_v_/F_m_ was strongly reduced). In conclusion, the effects of fluctuating light conditions on plant biomass are likely primarily determined by chlorophyll content, light absorption by the leaves and the rate at which photosynthetic biochemistry responds to changes in light intensity, which are the main determining factors of whole-plant photosynthesis.

### 4.2 Frequency of light fluctuations affects tomato morphology and physiology only at extreme amplitudes

In studies on light fluctuations published so far, a wide range of fluctuating light conditions were applied. However, studies that combine a number of frequencies and amplitudes of fluctuating light with comparable daily light integrals are still lacking (Morales and Kaiser, 2020). Therefore, in this study, we applied fluctuating light conditions with different amplitudes (200/0, 175/25, 125/75 and 100/100 µmol m^-2^ s^-1^) and different frequencies (hours, 30 minutes, minutes) while maintaining a constant DLI, to determine to which extent amplitudes and frequencies of fluctuations affect biomass, morphology and physiology. Light intensity fluctuations resulted in a reduction in shoot dry weight, primarily when light intensity switched between 200 and 0 μmol m^−2^ s^−1^. Our results showed that under these conditions, plant dry weight was more adversely affected when the frequency of fluctuations increased. When a minimum light level of 25 μmol m^−2^ s^−1^ was maintained, some plant traits were affected such as shoot biomass, chlorophyll content and Φ_PSII_, the extent to which depended on the frequency of light fluctuations. Our statistical model suggested that among all morphological and physiological traits that were affected by fluctuating light (**Table 2**, **Figures 2**, **3**, **5**), the interaction between frequency and amplitude was significant. This suggests that the effects of amplitude depended on the frequency of fluctuations, and vice versa. To the best of our knowledge, this is the first study in which the effects of frequency and amplitude of light fluctuations are combined, with the aim to determine which of these is the main determining factor for observed plant responses. Our results show that neither amplitude nor frequency can be considered more decisive for plant growth *per se*; instead, only extreme amplitudes of light fluctuations reduce plant biomass, and is aggravated under increased frequency of light fluctuations.

### 4.3 Severe reduction in plant biomass and chlorophyll content when switching lights on and off every minute might be caused by disturbed signal transduction

When lights were switched on and off every minute, leaf area, chlorophyll content, leaf development rate and plant biomass were significantly reduced. Furthermore, Φ_PSII_, F_v_/F_m_ and *A*_net_ were extremely low, showing that processes were disturbed to an extent that normal functioning and physiology were severely impaired. These symptoms suggest that the formation of some compounds central to plant functioning was severely disturbed, in turn impairing the plant’s ability to intercept light, photosynthesize, and grow. We do not know which processes were disrupted to lead to the observed – extreme – growth phenotype. Signal transduction pathways are inherently complex, and involve (at least) sensing by pigments or photoreceptors, signal transduction, gene expression, and protein formation, and any of these could have been disturbed under this specific light regime. However, we note that chlorophyll concentrations were strongly reduced, which may be a hint that chlorophyll biosynthesis was disturbed. One possible explanation for this may be reduced activity of the bZIP transcription factor ELONGATED HYPOCOTYL 5 (HY5) (Xiao et al., 2022). In darkness, HY5 is targeted for degradation while in light, HY5 accumulates in the nucleus (Osterlund et al., 2000; Hoecker, 2017; Lau et al., 2019; Ponnu et al., 2019; Ponnu and Hoecker, 2021). There, HY5 regulates the expression of thousands of genes involved in photomorphogenesis, photoprotection, root development, nutrient uptake, and biosynthesis of pigments such as anthocyanins and chlorophylls (Xiao et al., 2022; Wang et al., 2018; Van Gelderen et al., 2018; 2021; Sakuraba and Yanagisawa, 2018; Liu et al., 2018; Yan et al., 2023). It is unknown how long it takes for HY5 to accumulate in the nucleus and (up)regulate its target genes upon the transition from darkness to light. Thus one minute of illumination, followed by one minute of darkness, may be insufficient for HY5 to accumulate. Severely reduced HY5 accumulation may limit chlorophyll biosynthesis directly and/or limit the capacity of plants to reduce photoinhibition, eventually leading to the observed reduction in chlorophyll levels in the leaves. In Arabidopsis, it was shown that HY5 accumulation occurs under very low light intensities (<1 μmol m^−2^ s^−1^) and increases under increasing light intensities (Osterlund et al., 2000). This could explain why, when light fluctuated every minute between 175 and 25 μmol m^−2^ s^−1^, effects on biomass accumulation and most underlying processes were insignificant. Light fluctuations between 200 and 0 μmol m^−2^ s^−1^ at a frequency of 30 minutes also resulted in reduced plant biomass (**Figure 2A**) and chlorophyll content (**Figure 3A**), although much less severely than at a frequency of one minute. Possibly, the 30 minute light period is sufficient for HY5 to accumulate, but not up to levels that are realized under constant light. Measuring HY5 accumulation in leaves in future experiments may provide more insights in its role in plant responses to dynamic lighting strategies. Again, we would like to emphasize that besides the HY5 pathway described here, other pathways could be the reason for the observed phenotype under frequent changes between 200 and 0 μmol m^−2^ s^−1^. To identify target processes, future work could utilize screens of well-established mutants in model species, such as *Arabidopsis thaliana*.

### 4.4 Control strategies for lighting should be based on minimum light intensities to prevent adverse crop effects

Under conditions where light intensities in winter are insufficient to secure production and product quality, supplemental lighting is provided. A commonly used strategy is to switch off or dim the lighting at global radiation levels that exceed a pre-set threshold level (Dieleman et al., 2016; Mosharafian et al., 2021). However, more advanced control systems were designed with the aim to reduce the electricity consumption associated with lighting (Kjaer et al., 2012; Afzali et al., 2021; Serale et al., 2021; Kaiser et al., 2024). In general, these controls are based on sunlight predictions and aim for a minimal input of assimilation lighting, based on electricity costs and required values for the daily light integral (DLI), ETR or a calculated photosynthesis integral. These controls assume a fixed relationship between plant biomass accumulation and DLI. However, similar DLIs may result in differences in plant biomass accumulation, morphology and physiological traits at extreme light fluctuations within 24 hour (this study; Vialet-Chabrand et al., 2017; Bhuiyan and Van Iersel, 2021). When light fluctuations during the day were kept moderate, intelligent lighting control, balancing electricity prices and daily photosynthesis integral was shown to reduce energy costs without adversely affecting campanula dry weight when DLI was maintained (Kjaer et al., 2012). That implies that in strategies that control lighting not only DLI but also threshold levels for light intensity should be implemented. However, these data are only scarcely available. Questions that remain are what the threshold levels for light intensity and their maximum duration within 24 h are for different crops and crop stages. These threshold levels may also be affected by other environmental factors such as air temperature and CO_2_ concentration. Data available so far suggest that in lettuce, a minimum light level of 80 μmol m^−2^ s^−1^ should be maintained (Bhuiyan and Van Iersel, 2021), whereas in young tomato plants 25 μmol m^−2^ s^−1^ might be sufficient (this study). When implementing intelligent greenhouse climate control based on DLI and electricity prices, available knowledge on threshold levels for light intensities during the day should be incorporated.

## Data Availability

The raw data supporting the conclusions of this article will be made available by the authors, without undue reservation.

## Supplementary material

Supplementary Figure 1 Effect of light treatments (experiment 1) on the efficiency of photosystem II (Φ_PSII_) and electron transport rate (ETR) calculated based on chlorophyll fluorescence measurements every 15 minutes using the WALZ MICRO-PAM Monitoring System during 24 h for treatments **(A)** Cont 16h 100, **(B)** Hour 200/0, **(C)** 30 min 125/75, **(D)** 30 min 175/25, **(E)** 30 min 200/0 and **(F)** 1 min 200/0. Values of Φ_PSII_ in darkness (grey background) are equivalent to dark-adapted F_v_/F_m_. Data are the means of 2 repetitions, each existing of 3 biological replicates.

Supplementary Figure 2 Effect of light treatments (experiment 2) on the efficiency of photosystem II e (Φ_PSII_) and electron transport rate (ETR) calculated based on chlorophyll fluorescence measurements every 15 minutes using the WALZ MICRO-PAM Monitoring System during 24 h for treatments **(A)** Cont 16h 100, **(B)** Cont 8h 200, **(C)** Cont 16h 200, **(D)** 1 min 125/75, **(E)** 1 min 175/25 and **(F)** 1 min 200/0. Values of Φ_PSII_ in darkness (grey background) are equivalent to dark-adapted F_v_/F_m_. Data are the means of 2 repetitions, each existing of 3 biological replicates.

## References

[B1] Afzali S. Mosharafian S. Van Iersel M. W. Mohammadpour Velni J. (2021). Development and implementation of an IoT-enabled optimal and predictive lighting control strategy in greenhouses. Plants 10, 2652. doi: 10.3390/plants10122652, PMID: 34961123 PMC8703560

[B2] Bantis F. Smirnakou S. Ouzounis T. Koukounaras A. Ntagkas N. Radoglou K. (2018). Current status and recent achievements in the field of horticulture with the use of light-emitting diodes (LEDs). Sci. Hortic. 235, 437–451. doi: 10.1016/j.scienta.2018.02.058

[B3] Bhuiyan R. Van Iersel M. W. (2021). Only extreme fluctuations in light levels reduce lettuce growth under sole source lighting. Front. Plant Sci. 12. doi: 10.3389/fpls.2021.619973, PMID: 33584773 PMC7875872

[B4] Dieleman J. A. De Visser P. H. B. Meinen E. Grit J. G. Dueck T. A. (2019). Integrating morphological and physiological responses of tomato plants to light quality to the crop level by 3D modeling. Front. Plant Sci. 10. doi: 10.3389/fpls.2019.00839, PMID: 31354751 PMC6637845

[B5] Dieleman J. A. De Visser P. H. B. Vermeulen P. C. M. (2016). Reducing the carbon footprint of greenhouse grown crops: Re-designing LED-based production systems. Acta Hortic. 1134, 395–402. doi: 10.17660/ActaHortic.2016.1134.51

[B6] Genty B. Briantais J. M. Baker N. R. (1989). The relationship between the quantum yield of photosynthetic electron transport and quenching of chlorophyll fluorescence. Biochim. Biophys. Acta 990, 87–92. doi: 10.1016/S0304-4165(89)80016-9

[B7] Gitelson A. A. Merzlyak M. N. (1996). Signature analysis of leaf reflectance spectra: algorithm development for remote sensing of chlorophyll. J. Plant Physiol. 148, 494–500. doi: 10.1016/S0176-1617(96)80284-7

[B8] Hoecker U. (2017). The activities of the E3 ubiquitin ligase COP1/SPA, a key repressor in light signaling. Curr. Opin. Plant Biol. 37, 63–69. doi: 10.1016/j.pbi.2017.03.015, PMID: 28433946

[B9] Kaiser E. Kusuma P. Vialet-Chabrand S. Folta K. Liu Y. Poorter H.. (2024). Vertical farming goes dynamic: optimizing resource use efficiency, product quality, and energy costs. Front. Plant Sci. 2. doi: 10.3389/fsci.2024.1411259

[B10] Kaiser E. Matsubara S. Harbinson J. Heuvelink E. Marcelis L. F. M. (2018). Acclimation of photosynthesis to lightflecks in tomato leaves: interaction with progressive shading in a growing canopy. Phys. Plant 162, 506–517. doi: 10.1111/ppl.12668, PMID: 29125181

[B11] Kaiser E. Morales A. Harbinson J. Kromdijk J. Heuvelink E. Marcelis L. F. M. (2015). Dynamic photosynthesis in different environmental conditions. J. Exp. Bot. 66, 2415–2426. doi: 10.1093/jxb/eru406, PMID: 25324402

[B12] Kaiser E. Walther D. Armbruster U. (2020). Growth under fluctuating light reveals large trait variation in a panel of Arabidopsis accessions. Plants 9, 316. doi: 10.3390/plants9030316, PMID: 32138306 PMC7154841

[B13] Kalaji H. M. Schansker G. Ladle R. J. Goltsev V. Bosa K. Allakhverdiev S. I.. (2014). Frequently asked questions about *in vivo* chlorophyll fluorescence: practical issues. Photosynth. Res. 122, 121–158. doi: 10.1007/s11120-014-0024-6, PMID: 25119687 PMC4210649

[B14] Kimura H. Hashimoto-Sugimoto M. Iba K. Terashima I. Yamori W. (2020). Improved stomatal opening enhances photosynthetic rate and biomass production in fluctuating light. J. Exp. Bot. 71, 2339–2350. doi: 10.1093/jxb/eraa090, PMID: 32095822

[B15] Kjaer K. H. Ottosen C. (2011). Growth of chrysanthemum in response to supplemental light provided by irregular light breaks during the night. J. Am. Soc Hortic. Sci. 136, 3–9. doi: 10.21273/JASHS.136.1.3

[B16] Kjaer K. H. Ottosen C. O. Jørgensen B. N. (2011). Cost-efficient light control for production of two campanula species. Sci. Hortic. 129, 825–831. doi: 10.1016/j.scienta.2011.05.003

[B17] Kjaer K. H. Ottosen C. O. Jørgensen B. N. (2012). Timing growth and development of campanula by daily light integral and supplemental light level in a cost-efficient light control system. Sci. Hortic. 143, 189–196. doi: 10.1016/j.scienta.2012.06.026

[B18] Lau K. Podolec R. Chappuis R. Ulm R. Hothorn M. (2019). Plant photoreceptors and their signaling components compete for COP1 binding via VP peptide motifs. EMBO J. 38, e102140. doi: 10.15252/embj.2019102140, PMID: 31304983 PMC6745501

[B19] Liu C. C. Chi C. Jin L. J. Zhu J. Yu J. Q. Zhou Y. H. (2018). The bZip transcription factor HY5 mediates CRY1a-induced anthocyanin biosynthesis in tomato. Plant Cell Environ. 41, 1762–1775. doi: 10.1111/pce.13171, PMID: 29566255

[B20] Long S. P. Taylor S. H. Burgess S. J. Carmo-Silva E. Lawson T. De Souza A. P.. (2022). Into the shadows and back into sunlight: photosynthesis in fluctuating light. Ann. Rev. Plant Biol. 73, 617–648. doi: 10.1146/annurev-arplant-070221-024745, PMID: 35595290

[B21] Morales A. Kaiser E. (2020). Photosynthetic acclimation to fluctuating irradiance in plants. Front. Plant Sci. 11. doi: 10.3389/fpls.2020.00268, PMID: 32265952 PMC7105707

[B22] Morrow R. C. (2008). LED lighting in horticulture. HortSci. 43, 1947–1950. doi: 10.21273/HORTSCI.43.7.1947

[B23] Mosharafian S. Afzali S. Weaver G. M. van Iersel M. Velni J. M. (2021). Optimal lighting control in greenhouse by incorporating sunlight prediction. Comp. Elec. Agric. 188, 106300. doi: 10.1016/j.compag.2021.106300

[B24] Osterlund M. T. Hardtke C. S. Wei N. Deng X. W. (2000). Targeted destabilization of HY5 during light-regulated development of Arabidopsis. Nature 405, 462–466. doi: 10.1038/35013076, PMID: 10839542

[B25] Paradiso R. Proietti S. (2022). Light-quality manipulation to control plant growth and photomorphogenesis in greenhouse horticulture: the state of the art and the opportunities of modern LED systems. J. Plant Growth Regul. 41, 742–780. doi: 10.1007/s00344-021-10337-y

[B26] Ponnu J. Hoecker U. (2021). Illuminating the COP1/SPA ubiquitin ligase: fresh insights into its structure and functions during plant photomorphogenesis. Front. Plant Sci. 12. doi: 10.3389/fpls.2021.662793, PMID: 33841486 PMC8024647

[B27] Ponnu J. Riedel T. Penner E. Schrader A. Hoecker U. (2019). Cryptochrome 2 competes with COP1 substrates to repress COP1 ubiquitin ligase activity during Arabidopsis photomorphogenesis. Proc. Natl. Acad. Sci. U.S.A. 116, 27133–27141. doi: 10.1073/pnas.1909181116, PMID: 31822614 PMC6936435

[B28] Porcar-Castell A. Tyystjärvi E. Atherton J. van der Tol C. Flexas J. Pfündel E. E.. (2014). Linking chlorophyll a fluorescence to photosynthesis for remote sensing applications: mechanisms and challenges. J. Exp. Bot. 65, 4065–4095. doi: 10.1093/jxb/eru191, PMID: 24868038

[B29] Sakuraba Y. Yanagisawa S. (2018). Light signalling-induced regulation of nutrient acquisition and utilisation in plants. Semin. Cell Dev. Biol. 83, 123–132. doi: 10.1016/j.semcdb.2017.12.014, PMID: 29288799

[B30] Serale G. Gnoli L. Giraudo E. Fabrizio E. (2021). A supervisory control strategy for improving energy efficiency of artificial lighting systems in greenhouses. Energies 14, 202. doi: 10.3390/en14010202

[B31] Shinde P. Amelin M. (2019). A literature review of intraday electricity markets and prices. IEEE Milan PowerTech. 1–6. doi: 10.1109/PTC.2019.8810752

[B32] Van Gelderen K. Kang C. Li P. Pierik R. (2021). Regulation of lateral root development by shoot-sensed far-red light via HY5 is nitrate-dependent and involves the NRT2. 1 nitrate transporter. Front. Plant Sci. 12. doi: 10.3389/fpls.2021.660870, PMID: 33868355 PMC8045763

[B33] Van Gelderen K. Kang C. Paalman R. Keuskamp D. Hayes S. Pierik R. (2018). Far-red light detection in the shoot regulates lateral root development through the HY5 transcription factor. Plant Cell 30, 101–116. doi: 10.1105/tpc.17.00771, PMID: 29321188 PMC5810572

[B34] Vialet-Chabrand S. Matthews J. S. A. Simkin A. J. Raines C. A. Lawson T. (2017). Importance of fluctuations in light on plant photosynthetic acclimation. Plant Physiol. 173, 2163–2179. doi: 10.1104/pp.16.01767, PMID: 28184008 PMC5373038

[B35] Wang F. Wu N. Zhang L. Ahammed G. J. Chen X. Xiang X.. (2018). Light signaling-dependent regulation of photoinhibition and photoprotection in tomato. Plant Physiol. 176, 1311–1326. doi: 10.1104/pp.17.01143, PMID: 29146776 PMC5813521

[B36] Wang Q. Gao J. Chen J. Y. Tan X. M. Liu C. Y. Yu L.. (2024). Regulatory mechanism of a light-dependent protochlorophyllide oxidoreductase in chlorophyll biosynthesis and environmental adaptation. Technol. Agron. 4, 1–10. doi: 10.48130/tia-0024-0019

[B37] Xiao Y. Chu L. Zhang Y. Bian Y. Xiao J. Xu D. (2022). HY5: A pivotal regulator of light-dependent development in higher plants. Front. Plant Sci. 12. doi: 10.3389/fpls.2021.800989, PMID: 35111179 PMC8801436

[B38] Yan J. Liu J. Yang S. Jiang C. Liu Y. Zhang N.. (2023). Light quality regulates plant biomass and fruit quality through a photoreceptor-dependent HY5-LHC/CYCB module in tomato. Hortic. Res. 10 (12), uhad219. doi: 10.1093/hr/uhad219, PMID: 38077493 PMC10699845

[B39] Zhang Y. Kaiser E. Marcelis L. F. M. Yang Q. Li T. (2020). Salt stress and fluctuating light have separate effects on photosynthetic acclimation, but interactively affect biomass. Plant Cell Environ. 43, 2192–2206. doi: 10.1111/pce.13810, PMID: 32463133

[B40] Zhu X.-G. Ort D. R. Whitmarsh J. Long S. P. (2004). The slow reversibility of photosystem II thermal energy dissipation on transfer from high to low light may cause large losses in carbon gain by crop canopies: a theoretical analysis. J. Exp. Bot. 55, 1167–1175. doi: 10.1093/jxb/erh141, PMID: 15133059

