# Supplementary material for: Frequency of light fluctuations affects tomato morphology and physiology only at extreme amplitudes
Source: Front Plant Sci. 2025 Jul 8;16:1500197. doi: 10.3389/fpls.2025.1500197 (PMC12279503; doi:10.3389/fpls.2025.1500197)
Supplement: Supplementary Figure 1 — Effect of light treatments (experiment 1) on the efficiency of photosystem II (ΦPSII) and electron transport rate (ETR) calculated based on chlorophyll fluorescence measurements every 15 minutes using the WALZ MICRO-PAM Monitoring System during 24 h for treatments (A) Cont 16h 100, (B) Hour 200/0, (C) 30 min 125/75, (D) 30 min 175/25, (E) 30 min 200/0 and (F) 1 min 200/0. Values of ΦPSII in darkness (grey background) are equivalent to dark-adapted Fv/Fm. Data are the means of 2 repetitions, each existing of 3 biological replicates. [file DataSheet1.docx]

**Frequency of Light Fluctuations affects Tomato Morphology and Physiology only at extreme amplitudes**

**J. Anja Dieleman, Guido van Steekelenburg, Kees Weerheim, Elias Kaiser, Esther Meinen, Mark van Hoogdalem**

Supplemental information


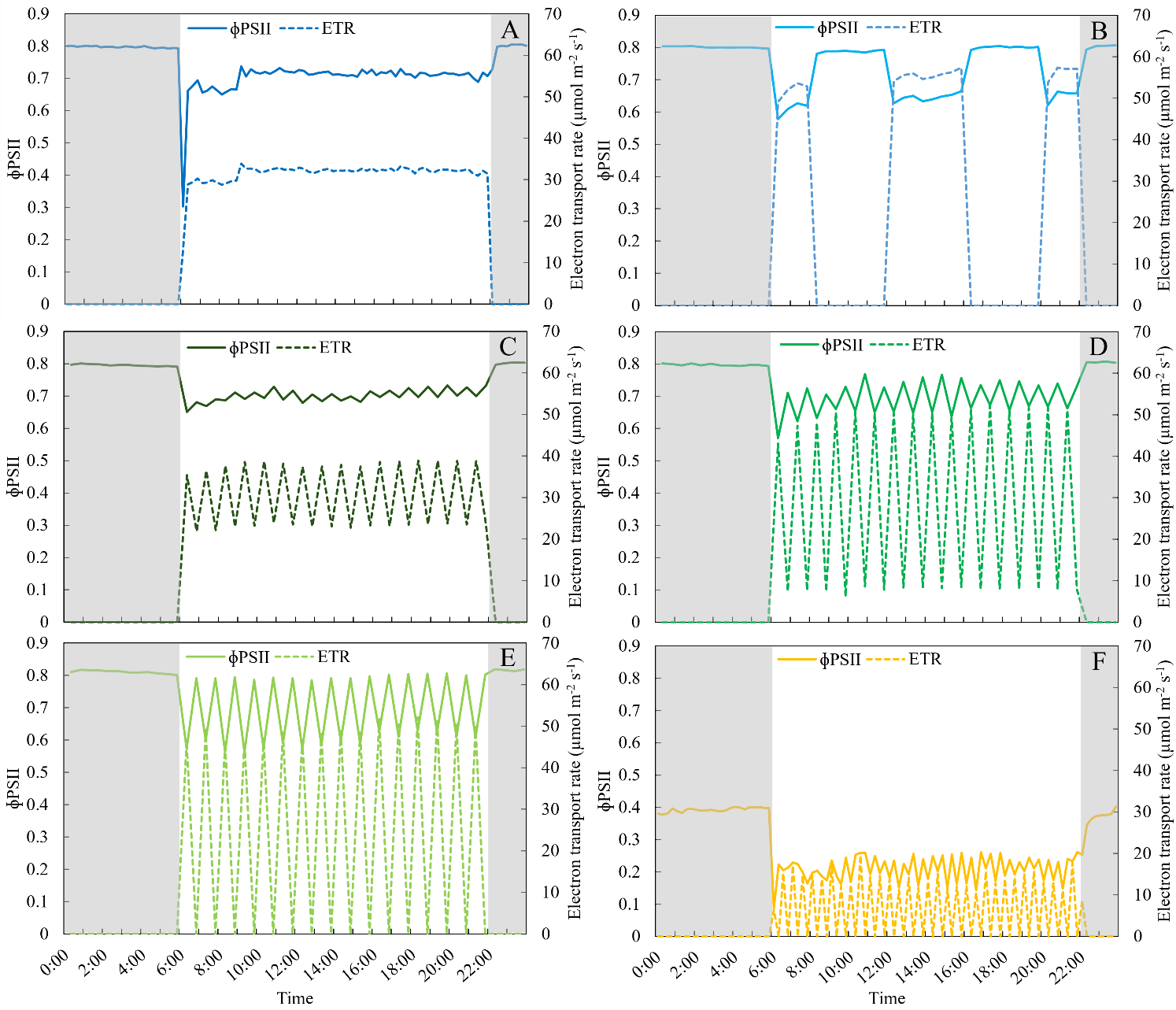


**SUPPLEMENTAL FIGURE 1│**Effect of light treatments (experiment 1) on the efficiency of photosystem II (ɸ_PSII_) and electron transport rate (ETR) calculated based on chlorophyll fluorescence measurements every 15 minutes using the WALZ MICRO-PAM Monitoring System during 24 h for treatments (A) Cont 16h 100, (B) Hour 200/0, (C) 30 min 125/75, (D) 30 min 175/25, (E) 30 min 200/0 and (F) 1 min 200/0. Values of ɸ_PSII_ in darkness (grey background) are equivalent to dark-adapted F_v_/F_m_. Data are the means of 2 repetitions, each existing of 3 biological replicates.


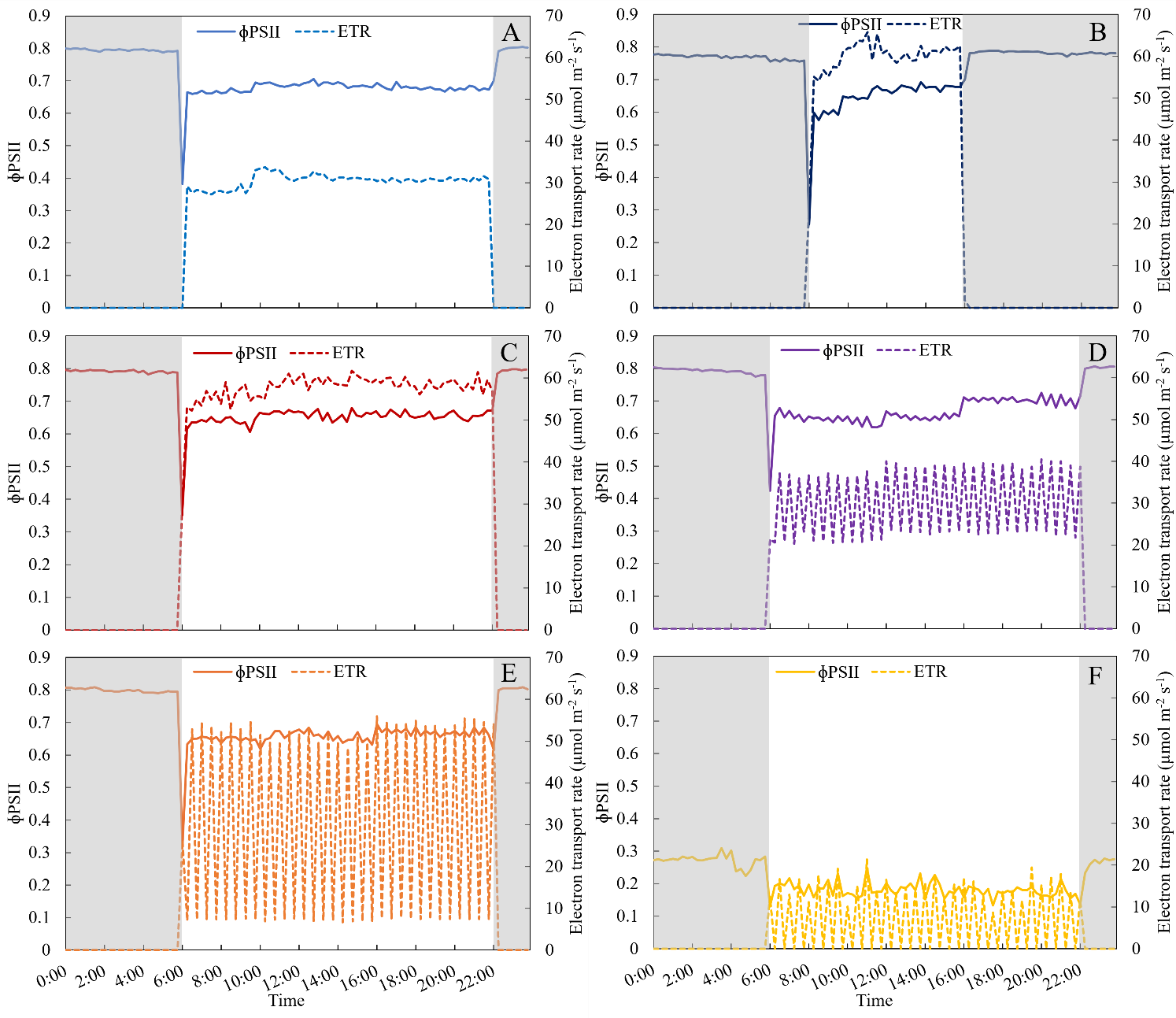


**SUPPLEMENTAL FIGURE 2│**Effect of light treatments (experiment 2) on the efficiency of photosystem II e (ɸ_PSII_) and electron transport rate (ETR) calculated based on chlorophyll fluorescence measurements every 15 minutes using the WALZ MICRO-PAM Monitoring System during 24 h for treatments (A) Cont 16h 100, (B) Cont 8h 200, (C) Cont 16h 200, (D) 1 min 125/75, (E) 1 min 175/25 and (F) 1 min 200/0. Values of ɸ_PSII_ in darkness (grey background) are equivalent to dark-adapted F_v_/F_m_. Data are the means of 2 repetitions, each existing of 3 biological replicates.
